# Fetal Alcohol Spectrum Disorders (FASD): an Approach to Effective Prevention

**DOI:** 10.1007/s40474-016-0101-y

**Published:** 2016-10-26

**Authors:** Sylvia Roozen, D. Black, G-J. Y. Peters, G. Kok, D. Townend, J. G. Nijhuis, G. H. Koek, L. M. G. Curfs

**Affiliations:** 1 Governor Kremers Centre, Maastricht University Medical Centre+, PO Box 5800, 6202 AZ Maastricht, The Netherlands; 2 Department of Work and Social Psychology, Maastricht University, Maastricht, The Netherlands; 3 European FASD Alliance, Landskrona, Sweden; 4 Faculty of Psychology and Education Science, Open University of the Netherlands, Heerlen, Netherlands; 5 Department of Health, Ethics & Society, Maastricht University, Maastricht, The Netherlands; 6 Department of Obstetrics & Gynaecology, Maastricht University Medical Centre+, Maastricht, The Netherlands; 7 Department of Internal Medicine, Division of Gastroenterology and Hepatology, Maastricht University + Medical Centre, Maastricht, The Netherlands; 8 Department of Genetics, Maastricht University Medical Centre+, Maastricht, The Netherlands

**Keywords:** Fetal alcohol syndrome, Fetal alcohol spectrum disorder(s), Health education, Prevention, Intervention, Prenatal alcohol exposure

## Abstract

**Purpose of Review:**

The objective of the current contribution is to propose an evidence-based, six-step approach to develop effective programs for prevention of fetal alcohol spectrum disorders.

**Recent Findings:**

Despite widespread campaigns aimed to reduce prenatal alcohol exposure, the number of affected children continues to be high. Current strategies to reduce prenatal alcohol exposure may be ineffective or counterproductive. However, proven principles of health promotion could be applied to reduce drinking in pregnancy. One such approach is Intervention Mapping (IM), a six-step procedure based on proven principles to change behaviors.

**Summary:**

FASD affects all communities and is an underestimated problem worldwide. Programs based on proven principles of behavior change are warranted. Program developers can use pre-existing protocols and strategies from evidence-based practice, such as Intervention Mapping. Developers who plan their preventive programs in a systematic and evidence-based manner increase the chances of success in reducing prenatal alcohol exposure and FASD.

## Introduction

Consumption of ethyl alcohol can have negative impact on health and quality of life. As alcohol consumption is part of an accepted way of life in many countries, the healthcare problems and costs are tremendous. Many persons are unaware of the toxic effect of alcohol and its metabolites on different organs of the body. Children, young adults, women, and in particular pregnant women are very vulnerable to the effects of alcohol that can have lifelong consequences. This is especially the case with fetal alcohol spectrum disorders (FASD). FASD is an umbrella term for a range of birth defects caused by prenatal exposure to ethyl alcohol. Alcohol results in mild to severe damage to the development of the unborn baby [1–3]. This damage leads to lifelong physical, behavioral, and cognitive disabilities. Depending on the nature and severity of the damage, the following diagnoses under the FASD umbrella can be given: fetal alcohol syndrome (FAS), partial fetal alcohol syndrome (pFAS), alcohol-related neurodevelopmental problems (ARND), alcohol-related birth defects (ARBD), or neurobehavioral disorder-prenatal alcohol exposed (ND-PAE) [1, 4–8]. FASD is a 100 % preventable disorder, as alcohol consumption during pregnancy can be avoided. FASD is therefore one of the most important preventable forms of non-genetic birth defects associated with mental retardation [9–12]. This paper provides an overview of the current state of FASD in relation to diagnostic procedures, prevalence, and prevention, as well as making recommendations for the way forward in FASD prevention.

## Diagnostics and Prevalence

FAS, first named in 1973, comprises several birth defects such as growth deficiency (pre- and/or postnatal), facial dysmorphology (short palpebral fissures, a thin upper lip, smooth philtrum), or central nervous system dysfunction (structural, neurological, or functional) [13]. In cases where the full range of birth defects is not seen, other diagnostic terms are used, and these are currently grouped under the umbrella term FASD. Epidemiological studies have been conducted in countries such as Australia, Canada, Italy, South Africa, and the USA, with FASD prevalence prediction intervals ranging from 0 to 176.77 per 1000 live births [14]. These estimates are not easy to interpret as they are influenced by factors such as the target group from which a sample was taken and the guidelines applied for diagnosis. Indeed, the difficulty of determining the prevalence goes hand-in-hand with the difficulty of diagnosing the disorders.

Several initiatives have been undertaken to achieve a consensus on establishing diagnoses within the spectrum of FASD [15]. The Institute of Medicine (IOM) of the USA published a first consensus in 1996 which was revised in 2005 and recently updated [11, 16, 17]. Disagreement about the strictness of the diagnostic criteria and their practical application remained. Between 1997 and 2004, Astley and Clarren developed a 4-digit diagnostic code to improve the objectivity of the diagnosis [18]. In 2005, the Canadian guidelines were published, which are considered to be midway between the IOM and the 4-digit criteria [19]. In 2014, new diagnostic criteria for ND-PAE were developed and proposed for further evaluation in the fifth edition of the Diagnostic and Statistical Manual [6, 7]. However, the IOM guideline is currently the most commonly applied tool [14, 17].

## Prevention

Many approaches have been taken in the attempt to prevent FASD. Some approaches are targeted to the general public, including labels on drink containers, large-scale distributions of posters, and flyers or media campaigns, such as the international campaigns taking place annually on September 9, International FASDay [20]. Other approaches target professionals, for example, accredited training sessions for physicians and midwives. Still other programs focus on women at risk of an alcohol-exposed pregnancy or on pregnant women. Such programs range from brief interventions to intensive accompaniment during the pregnancy [21].

In many cases, there has unfortunately been inadequate evaluation of the effectiveness of the intervention. For example, many of the campaigns targeted to the public carry out no evaluation at all, or the evaluation is based on the response to questions about awareness of the risks associated with prenatal exposure to alcohol. It is often assumed that heightened awareness of the risks will lead to stoppage of drinking during pregnancy, yet this may not be the case.

A comparison with campaigns influencing smoking behavior can be illuminating. For years, many different attempts have been made to address smoking behavior, both effective and ineffective. An ineffective approach is, for example, fear appeal images and texts which are printed on cigarette packages. Research shows that fear appeal interventions are not effective in changing behavior [22, 23]. For the development of measures to prevent alcohol consumption during pregnancy, we can learn from these studies and the use of evidence-based strategies. Up to now, there has been little use of evidence-based strategies to reduce alcohol consumption during pregnancy [21]. The use of randomized control trials is an exception rather than the rule.

Given the serious consequences, prevention of prenatal exposure to alcohol is urgently needed. In order to go forward, several recommendations can be made.

## Recommendations

Prevention is the key in FASD whereby evidence-based practice is of utmost importance. First, it is essential to have a good understanding of what is known and what is still unknown about FASD. Evidence-based practice entails an integration of scientific evidence and clinical expertise to form the basis for FASD prevention and management. An iterative approach can be used for the process of problem identification to solving the problem. In this effort, pre-existing protocols and strategies can be used, such as Intervention Mapping (IM) [24, 25]. Intervention Mapping is a planning approach that is based on using theory and evidence as foundations for taking an ecological approach to assessing and intervening in health problems and engendering community participation [24]. IM offers a methodology to systematically develop, implement, and evaluate health promotion programs.

**Fig. 1:**
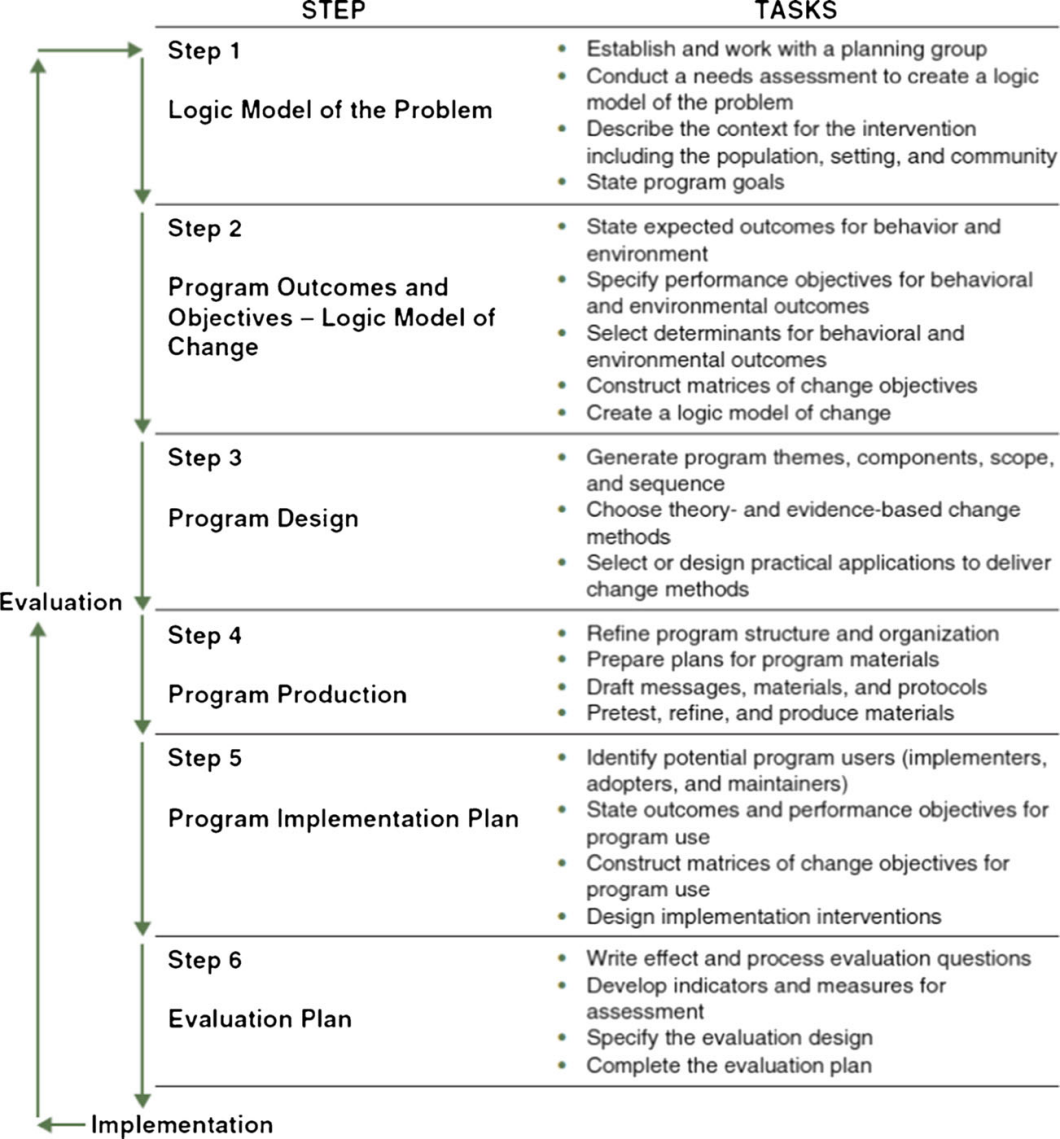
Intervention Mapping protocol [24]

Each step of Intervention Mapping comprises several tasks (Fig. 1). The completion of the tasks included in a step creates a product that is the guide for the subsequent step. Completion of all steps creates a blueprint for designing, implementing, and evaluating an intervention based on a foundation of theoretical, empirical, and practical information. Although Intervention Mapping comprises six steps, the process is iterative rather than completely linear. Program developers move back and forth between tasks and steps as they gain new information and perspective. However, the process is also cumulative; planners base each step on the previous steps, and inattention to a step can jeopardize the potential effectiveness of later steps.

These six steps could be applied to the prevention of prenatal alcohol exposure and FASD as follows.

Step 1. Develop a logic model of the problem based on a needs assessment.

The first step in development of a systematic evidence-based health promotion program is to conduct a needs assessment or problem analysis by identifying what, if anything, needs to be changed and for whom. Planners need to get a good understanding of the nature, extent, and causes of the health problem before starting to develop a program. The following questions may apply. Is FASD a worldwide problem? The recently conducted meta-analyses of global FASD prevalence estimates can assist [14]. Data was only available for a limited number of countries with FASD prevalence estimates ranging from 0 to 176.77 per 1000 live births. Prevalence studies varied considerably in terms of their methodology, so caution is warranted in interpreting the results; however, one can conclude that FASD is indeed a worldwide problem that merits more attention. As FASD is caused by prenatal alcohol exposure, the question arises what maternal behaviors are related to FASD. There is no consensus on what can be understood under harmful behavior and the desired behavior among pregnant women. Some studies report that heavily drinking pregnant women (“binge drinking”) are more at risk of having a child with FASD [26]. Other studies report that mild to moderate amounts of alcohol consumption during pregnancy can already have adverse effects on the outcome of the unborn child [27]. Therefore, various international organizations such as the World Health Organization (WHO), American Academy of Pediatrics (AAP), National Institute of Health (NIH), National Institute on Alcohol Abuse and Alcoholism (NIAAA), and British Medical Association (BMA) conclude that there is no known safe amount of alcohol to consume while pregnant [11, 12, 28, 29]. To elicit changes in behavior, it is, however, important to provide insight into the exact behaviors, as well as the determinants and environmental factors which influence these behaviors. Once this has been studied, it is essential to determine who the highest at-risk target group is. For FASD, this could be alcohol-dependent pregnant women, women who are planning a pregnancy or women who potentially might get pregnant. Furthermore, who are the stakeholders involved who can contribute to the development and implementation of the program [30]? The selection of important stakeholders is not straightforward, but stakeholders potentially concerned with FASD include the following: parents (biological and adoptive parents), persons affected by FASD, the alcohol industry, government (various departments), healthcare professionals, researchers, and policy makers. Finally, what types of stigma play a role and need to be considered while developing a program? Are there any further ethical considerations to address [31, 32] ?

On the basis of the results of this first problem analysis, planners continue to step 2 to determine the program outcomes and objectives that should lead to a decrease of the health problem.

Step 2. State program outcomes and objectives—a logic model for change.

The second step is to create what are known as “matrices of change objectives” by combining behaviors and subsidiary behaviors with behavioral determinants to identify which determinants and beliefs should be targeted by the intervention [33]. This step is not only conducted for the behaviors of the at-risk target group, but repeated for behaviors required by relevant stakeholders (e.g., midwives, general practitioners). The analyses of relevant psychological determinants and environmental factors (both of which become desired intervention outcomes) are based both on psychological theory and empirical evidence. Moreover, relevant stakeholders are consulted and involved.

Step 3. Develop the program plan, including scope, sequence, change methods, and practical applications.

The third step is to select theory-based behavior change methods that match the determinants into which the identified sub-determinants aggregate and translate these into practical applications that satisfy the parameters for effectiveness of the selected methods [34]. For example, a study by France and colleagues (2014) developed messages to increase women’s intention to abstain from alcohol while pregnant [35]. The authors combined risk information and self-efficacy messages to achieve the goal of intention to abstain from alcohol while pregnant. While risk information alone was effective in increasing intention not to drink, theory suggests that threatening messages alone may not change actual behavior. The capacity to change behavior depends also on self-efficacy (one’s belief in the ability to succeed). Risk can be a motivator for changing behavior when the message is focused on increasing or improving self-efficacy [24].

Step 4. Produce the intervention, including program materials and messages.

Once all components of the program have been developed, these can be integrated into one coherent program. One example is the brochure corresponding with the publication by France and colleagues [35]. The fourth step is therefore based on developing plans for pre-testing the program materials together with the planning group (including the target population).

Step 5. Plan program use, including adoption, implementation, and maintenance.

The fifth step is to plan for adoption, implementation, and sustainability of the program. Once a program has been developed, this in itself will not guarantee effectiveness. The program users and implementers need to be identified. The most obvious and important implementers for FASD prevention are healthcare professionals in fields related to pregnancy. Analysis of the stakeholders from the first step can assist in the choice of implementers, as not all relevant stakeholders are motivated or in a position to carry out a program. The process and considerations for adoption, implementation, and sustainability of programs are often not reported.

Step 6. Develop an evaluation plan.

The sixth step is to generate an evaluation plan to measure program effectiveness. For each of the objectives from step 2, a measurable outcome should be determined. Then, the planners specify the evaluation design to conduct effect and process evaluations.

## Conclusions

FASD is an important health problem which affects individuals and their families worldwide. FASD is caused by prenatal alcohol exposure and is therefore, theoretically, entirely preventable. Development of effective interventions, however, is complex, and if key factors are overlooked, an ineffective or counterproductive program may be the result. Program developers can use pre-existing protocols and evidence-based strategies, such as the Intervention Mapping approach described here.
